# Application of Two-Stage Cultivation for Exploring the Nutritional Requirements for Sporulation of Three Biocontrol Fungi

**DOI:** 10.1155/2015/682839

**Published:** 2015-01-29

**Authors:** Li Gao

**Affiliations:** State Key Laboratory for Biology of Plant Disease and Insect Pests, Institute of Plant Protection, CAAS, Beijing 100193, China

## Abstract

Fungicide was an important part in mycopesticides, which play an important role in pest management, while their mass production and commercialization faced problem. We found that the nutrition for mycelia growth and sporulation differences a lot. So, we developed “two-step method” to define the nutrition for sporulation in this paper. The results indicated that the novel method led to a great increase of spore yields for *Beauveria bassiana* (IBC1201), *Lecanicillium lecanii* (CA-1-G), and *Pochonia chlamydosporia* (HSY-12-14), respectively, of about 100, 2, and 16 times and, also reduced the cycle of mass production to 1/3 compared with common time for culturing.

## 1. Introduction

Fungal biopesticides constitute an important part of microbial pesticides and play a crucial role in the biocontrol of plant diseases and pests. According to incomplete statistics, more than 30 species (58 products) of fungal biopesticides have been registered in the world [1]. Growth and reproduction of biocontrol fungi guarantee their completion of life and infection cycle, which are key steps in the industrialization of fungal biopesticides.

Spores are a major component of fungal biopesticides. Mass production of spores is a key factor restricting the application of fungal biopesticides. Optimal nutritional requirements are necessary conditions for mass production of fungal spores. Knowing the optimal conditions of biocontrol fungi for sporulation also helps us to understand their nutritional requirements under natural conditions, allowing the fungi to better grow and thereby fulfill the protective function in field [2], even though the efficiency of spores maybe related to the stress tolerance [3, 4].

Presently, liquid-solid two-phase fermentation is commonly used for spore production of fungal biopesticides worldwide. In the process of two-phase fermentation, liquid fungal culture is inoculated into solid substrates (e.g., rice husk or wheat bran) for sporulation after a liquid-state fermentation step. Because sporulation in solid-state fermentation generally involves shallow pan cultivation, mass production of spores requires substantial space. The process of solid-state fermentation takes five to ten days, accounting for a long production cycle and very high cost. Additionally, the sporulation rate in solid-state fermentation is relatively low (4–12%), producing 88–96% of wastes. It remains difficult to achieve economically successful industrialization exploitation via the liquid-solid two-phase production process, which substantially constrains the application of fungal biopesticides.

Carbon (C) source, nitrogen (N) source, vitamins, minerals, and nutrients are indispensable materials and energy sources for the growth of fungi. In a typical fungal cell, C and N elements, respectively, account for 50% and 5% of dry weight. A considerable amount of energy is obtained with oxidation of carbohydrates as the sole source. Vitamins and minerals are also essential to normal metabolism and physiological functions in cells. Only with certain nutritional supplies, the fungus can grow, reproduce, and complete the life cycle. Mass production of fungal spores, which can only be achieved based on a full understanding of the optimal conditions for vegetative growth, will promote the industrialization process of fungal biopesticides.

Many scholars have recognized the importance of nutrition research to the industrial production of fungal spores for biopesticides [5–11]. The biological characteristics of biocontrol fungi regarding colonization, dynamics, activity, and virulence to target pests in the natural environment are also restricted by nutritional conditions. It has been demonstrated that fungal spores produced under different nutritional conditions have varying levels of virulence to a target pest [12]. Therefore, to master the nutritional requirements of biocontrol fungi is a foundation for the industrialization of fungal biopesticides as well as a key step for effective application of the products.

In a previous study, we systematically tested C sources, N sources, C concentrations, and C/N ratios suitable for mycelial growth and sporulation in several biocontrol fungi [13, 14]. Each test fungus had large differences in the nutritional conditions suitable for mycelial growth and sporulation, indicating that the fungus had varying nutrients at different growth stages. Thus, there is great necessity to study the difference in nutritional requirements of biocontrol fungi in relation to growth stage. Relevant work can help meet the needs of biocontrol fungi from the physiological point of view, further to achieve cost-effective industrial production of fungal biopesticides.

Previous research has exclusively adopted conventional one-stage cultivation, which determines spore yield after vegetative growth on the same medium and thus can not truly reflect the nutritional requirements of the culture for sporulation. To address this issue, we developed a method called two-stage cultivation for better defining of the nutritional requirements for sporulation of biocontrol fungi [15]. The test fungi were cultured on a basal medium for four days (C source, N source, C concentration, and C/N ratio suitable for vegetative growth) and then transferred to specific test media for sporulation (based on suitable C and N sources with C concentration and C/N ratio adjusted for sporulation) for another four days of incubation. The optimal combination of relevant nutritional factors for sporulation was then screened out by considering the spore yield.

## 2. Materials and Methods

### 2.1. Fungal Strains

The biocontrol fungi included entomogenous* Beauveria bassiana *(IBC1201),* Lecanicillium lecanii* (CA-1-G), and nematophagous* Pochonia chlamydosporia* (HSY-12-14, Table 1). All these isolates were cultured in the Institute of Microbiology, Chinese Academy of Sciences.

### 2.2. Chemical Reagents

Analytical grade reagents were purchased from different Chinese companies. MnSO_4_·H_2_O and dehydrated CaCl_2_ were purchased from Beijing Yili Fine Chemicals Co., Ltd., KCl and soy peptone were purchased from Nanjing Reagent Co., Ltd., and Na_2_MoO_4_·2H_2_O, MgSO_4_, FeSO_4_, K_2_HPO_4_, ZnSO_4_·7H_2_O, and H_3_BO_4_ were purchased from Beijing Chemical Reagent Plant. CuSO_4_·5H_2_O was purchased from Beijing Shuanghuan Chemical Reagents Plant. Powdered agar was purchased from Shanghai Chemical Reagent Station.

### 2.3. Preparation of Culture Media

Basal medium was prepared with soy peptone (8 g/L) as C source and sucrose (0.33 g/L) as N source. The medium contained (per liter): 19.002 g sucrose, 4.059 g peptone, 1.000 g K_2_HPO_4_, 0.500 g KCl, 0.500 g MgSO_4_, 0.010 g FeSO_4_, and 13.000 g agar. Test media were formulated based on suitable C concentrations, C/N ratios, C sources, and N sources (Table 1) for sporulation that were screened out by single-factor experiments. The sporulation media were prepared with different combinations of C and N sources, in which the contents of other components remained unchanged (Table 1).

### 2.4. Preparation of Other Materials

New Petri dishes were prepared by soaking in 0.05% (v/v) hydrochloric acid for 24 h, followed by rinsing thrice with clear water and once with distilled water. The glassware was air-dried and then sterilized by dry heat at 180°C for 2-3 h before use. Cellophane membranes punched into 3.5 mm round pieces were autoclaved at 121°C for 30 min and weighted aseptically using a precision electronic balance (0.001 g). Basal medium was autoclaved under the same conditions and aseptically dispensed in 10 mL aliquots to clean Petri dishes. After cooling and solidification of the agar, round cellophane membranes were placed on the agar surface in a triangle pattern. These plates were kept standing for two days on an ultra-clean bench for evaporation of free water before inoculation.

### 2.5. Inoculation and Incubation

Spore suspensions (1 × 10^5^ spores/mL) were prepared in 50 mL centrifuge tubes containing 5 mL of autoclaved 0.50% (V/V) Tween 80 solution [11]. The prepared spore suspensions (0.5 *μ*L aliquots) were aseptically pipetted to the center of round cellophane membrane (with no free water) on basal medium. Each treatment was repeated independently three times. The inoculated plates were incubated at room temperature for 24 h and then wrapped with Parafilm, incubated at room temperature for three days. Cellophane membranes covered with fungal colonies were then transferred using a sterile pincet to the test media for sporulation. The inoculated plates were incubated under the same conditions for four days (two-stage cultivation). The corresponding cultures grown on basal medium for eight consecutive days were used as controls (one-stage cultivation).

### 2.6. Determination of Spore Yield

Cellophane membranes carrying fungal colonies were carefully taken and placed into 50 mL centrifuge tubes containing 10 mL of Tween 80 solution. The suspensions were vortexed for 3–5 min and then loaded onto a hemocytometer to count the number of spores by light microscopy (×40 magnification). Each treatment was repeated thrice.

### 2.7. Statistical Analysis

The experimental data were statistically analyzed by SAS 8.0 (SAS Institute Inc., Cary, NC, USA).

## 3. Results

Table 2 shows that, during two-stage cultivation,* B. bassiana* IBC1201 had significantly higher spore yields on the test media than on the control medium (35.00 × 10^5^ spores/mL; C conc. = 8 g/L, C/N ratio = 24 : 1). The spore yield of strain IBC1201 remained relatively high with glucose as the C source in combination with a variety of N sources. This result indicated that glucose is a suitable C source for sporulation of this fungus. The spore yield was highest with the combination of cellobiose + urea (3032.67 × 10^5^ spores/mL), which was approximately 100-fold that on the control medium. The spore yield with the combination of sucrose + yeast extract came second (2823.00 × 10^5^ spores/mL), approximately 80-fold that on the control medium.

In the two-stage cultivation of* L. lecanii* CA-1-G, the spore yield on the test media with the combination of glucose + urea was approximately 1.5-fold that on the control medium. Additionally, the spore yields of strain CA-1-G on the test media with the combinations of trehalose + urea (915.00 × 10^5^ spores/mL), trehalose + soy peptone (900.00 × 10^5^ spores/mL), and glucose + soy peptone (929.00 × 10^5^ spores/mL) were approximately 3-fold that on the control medium. The spore yields of strain CA-1-G achieved on the test media with the combinations of trehalose + yeast extract and glucose + yeast extract had minor differences from that on the control medium.

During two-stage cultivation with suitable C concentration (8 g/L) and C/N ratio (10 : 1) for sporulation,* P. chlamydosporia* HSY-12-14 had significantly higher spore yields on the test media with different combinations of C and N sources than on the control medium. The best result was achieved from the combination of sucrose + soy peptone (359.00 × 10^5^ spores/mL), that is, approximately 30-fold that on the control medium (C concentration = 8 g/L, C/N ratio = 24 : 1; 13.00 × 10^5^ spores/mL). The spore yields of strain HSY-12-14 with other combinations of C and N sources were only 4-fold that on the control medium.

## 4. Discussion

### 4.1. Implications of Two-Stage Cultivation

This study systematically assessed the effects of nutritional conditions (30 C sources, 19 N sources, and 15 C conc. and C/N ratios) on mycelial growth and sporulation of four biocontrol fungi. According to the experimental results, different combinations of C and N sources suitable for mycelial growth in liquid culture and sporulation in solid culture were identified. The two sets of media had large differences in nutritional composition, indicating that the test strains required varying types of C and N sources when cultured under different conditions.

Nutritional regulation is an important approach to further obtain the biomass of target microorganisms at specific growth stage (mycelium or spores). Two-stage cultivation is processed based on the master of physiological characteristics of target microorganisms and thereby can achieve stable and rapid production of the biocontrol strains. On the contrary, conventional cultivation (one-stage) takes no into account the specificity of nutritional requirements for the same strain or the changes in the type and amount of nutrients required by the strain at different growth stages. This is despite that relevant information is of great importance to the mass production of biocontrol strains and directly relates to the biomass yield, the length of production cycle, and the cost of production.

In the present study, all the test fungi were grown on basal medium and supplied with the nutrients within the first four days of cultivation. Cultures of the same strain were incubated under the same condition to achieve consistent growth and then transferred to test media with suitable C concentration and C/N ratio but different combinations of C and N sources for sporulation. The control treatment was grown on basal medium for eight consecutive days. The idea is to supply specific nutrients suitable for growth and sporulation at two different growth stages, respectively, which can not only obtain more biomass of target strains but also save resources and reduce costs. While reflecting the physiological needs of the four test fungi, the results provide a theoretical basis for better solving the problems regarding mass production.

### 4.2. Improvement of Spore Yield

Among the test strains, most produced far less spores after eight consecutive days of growth on the control medium (C conc. = 8 g/L, C/N ratio = 24 : 1; one-stage cultivation) than after four days of growth on the control medium and another four days of growth on the test media with different combinations of C and N sources for sporulation (two-stage cultivation). For example, when* B. bassiana *IBC1201 was grown with suitable C concentration (4 g/L) and C/N ratio (5 : 1), the spore yield in two-stage cultivation using the test media with different combinations of C and N sources was significantly higher than that in one-stage cultivation using the control medium (35.00 × 10^5^ spores/mL). The highest spore yield was obtained from the combination of cellobiose + urea (3032.67 × 10^5^ spores/mL), which was approximately 100-fold that on the control medium. The spore yield with the combination of sucrose + yeast extract was second highest (2823.00 × 10^5^ spores/mL), approximately 80-fold that on the control medium.

During two-stage cultivation of* L. lecanii *CA-1-G, its spore yield on the test media increased or decreased with different combinations of C and N sources compared to that on the control medium. The spore yield was highest on the test media with the combinations of cellobiose or glucose + soy peptone and cellobiose + urea, approximately 3-fold that on the control medium. As for* P. chlamydosporia* HSY-12-14, the spore yield in two-stage cultivation with the combination of sucrose and soy peptone was approximately 16-fold that on the control medium.

Although the two sets of media had identical components, two-stage cultivation employed the test media with a different C/N ratio of 10 : 1 at the sporulation stage, whereas one-stage cultivation used an unchanged C/N ratio of 24 : 1 (8 g/L) throughout the fermentation process. Therefore, the test strains commonly required varying C concentrations, C/N ratios, and combinations of C and N sources in relation to growth stages.

### 4.3. Shortening of the Production Cycle

Presently, production of fungal biopesticides mainly uses liquid-solid two-phase fermentation. In this process, the level of fermentation liquid is generally high. Solid-state substrates include grains such as rice, barley, and even some plant residues or wastes such as leaves and seeds. However, solid-state fermentation adopts shallow pan (or plastic bag) cultivation and thus requires substantial space, accounting for a low sporulation rate, 3–12% only. By comparison, two-stage cultivation can reduce the production space on the basis of increasing production yield. Its whole production process only takes eight days, which significantly shortens the production cycle compared to the conventional method that takes fifteen to twenty days. In short, this study is an exploration of the nutritional needs for biocontrol fungi and the obtained results provide a reference for large-scale production of fungal biopesticides.

### 4.4. Individual Differences among Biocontrol Fungi

For some strains, two-stage cultivation significantly improved the spore yield, such as* B. bassiana* IBC1201. In some cases, the optimal combination of C and N sources for sporulation was the same as that of the control medium, despite the changes in C concentration and C/N ratio, such as* P. chlamydosporia* HSY-12-14. During two-stage cultivation, the test strains had increased spore yields with certain combinations of C and N sources but decreased spore yields with other combinations compared to the yield on the control medium, such as* L. lecanii* CA-1-G. Hence, there is necessity to systematically study the nutritional requirements of individual biocontrol fungi, in order to improve the sporulation rate in large-scale production.

In the production of contamination- and pollution-free biocontrol strains, stable-yield, high-efficiency, and low-cost products are required to have strong efficacy and high virulence in practical applications. From production to actual biocontrol, a variety of factors directly affect the virulence of the products and their biocontrol effects, including nutritional conditions in relation to cultivation stage under study in this work. For example, different types and amounts of nutritional requirements can influence spore shape and virulence of biocontrol fungi [16], the same as packaging materials, packaging process, and product preservation during production of fungal biopesticides. In field applications, the above features are restricted by environmental factors such as temperature, humidity, and trace elements, which act through influencing spore germination, mycelial colonization, and reproduction.

Drought tolerance of spores has been an important issue that restricts the biocontrol efficiency of fungal biopesticides. The use of trehalose as the sole C source or one of the C sources (some strains can not grow well on the sole C source of trehalose) can greatly improve drought resistance of fungal spores in biocontrol fungi, thereby to a large extent guarantee the stable biocontrol efficiency of fungal biopesticides [3, 4]. Some N source can promote the formation of chlamydospores [17], while certain nutrients promote the formation of small sclerotia in some fungal strains [16]. All relevant factors can directly affect the control effect of fungal biopesticides in practical applications. Therefore, study is needed to further address a series of questions in order to provide the most reasonable culture method. Detailed study on the influences of nutritional and environmental factors on the growth and sporulation of biocontrol fungi (especially on spore yield and virulence) still has great reference value for research and development of fungal biopesticides [18].

## Figures and Tables

**Table 1:** Carbon concentrations and C/N ratios for sporulation.

Isolates	Host	Collection places	C/N ratio	Carbon concentration (g/L)	Carbon sources	Nitrogen sources	Minerals and vitamins
*B. bassiana* (IBC1201)	Locust	Tianjin, China	5 : 1	4	Sucrose, glucose, cellobiose	Yeast extract, urea, soy peptone	ZnSO_4_·7H_2_O 50 mg/L, CuSO_4_·5H_2_O 50 mg/L, CaCl_2_ g/L, MnSO_4_·H_2_O 5 mg/L

*L. lecanii* (CA-1-G)	*Myzus persicae *	Fujian, China	5 : 1	4	Glucose, trehalose, cellobiose	Yeast extract, soy peptone, urea	CuSO_4_·5H_2_O 10 mg/L, ZnSO_4_·7H_2_O 50 mg/L, CaCl_2_ 1 g/L, H_3_BO_4_ 5 mg/L, Na_2_MoO_4_·2H_2_O 5 mg/L

*P. chlamydosporia* (HSY-12-14)	*Meloidogyne incognita *	Hainan, China	10 : 1	8	Sucrose, maltose	NaNO_3_, urea	ZnSO_4_·7H_2_O 50 mg/L, H_3_BO_4_ 50 mg/L, MnSO_4_·H_2_O 50 mg/L

**Table 2:** Combination of carbon and nitrogen sources for sporulation by two-stage cultivation.

Tryptone	Yeast extract	Soy peptone	Urea	NaNO_3_	CK	LSD
IBC1201						
Cellobiose	1914.30d	642.73g	3032.67a			
Sucrose	2823.00b	580.00h	327.27i			
Glucose	1060.30f	2078.73c	1560.00e		35.00j	40.56
CA-1-G						
Trehalose	350.00bc	900.00a	915.00a			
Glucose	158.00c	929.00a	580.73b		303.80b	391.13
HSY 12-14						
Sucrose		359.00a	47.72b	47.00b		
Maltose		36.33c	47.72b	36.33c	13.00d	3.76

Values are means of three replicates; values in the same column followed by the same letter are not significantly different (LSD, *P⩽* 0.05).
